# A retrospective analysis of emergency hysterectomy intervention strategy in obstetrics

**DOI:** 10.12669/pjms.38.3.5335

**Published:** 2022

**Authors:** Zhuanji Fang, Huale Zhang, Shuisen Zheng, Lingling Weng, Jianying Yan

**Affiliations:** 1 Zhuanji Fang, MD. Department of Obstetrics, Fujian Maternity and Child Health Hospital, Affiliated Hospital of Fujian Medical University; No. 18, Daoshan Road, Fuzhou, 350000, Fujian Province, P.R. China; 2 Huale Zhang, MD. Department of Obstetrics, Fujian Maternity and Child Health Hospital, Affiliated Hospital of Fujian Medical University; No. 18, Daoshan Road, Fuzhou, 350000, Fujian Province, P.R. China; 3 Shuisen Zheng, MD. Fujian Medical Univeristy, 1 XueYuan Road, Fuzhou, 350000, Fujian Province, P.R. China; 4 Lingling Weng, MD. Department of Obstetrics and Gynecology, Ningde People’s Hospital, Bayiwu West Road, Ningde, 352000, Fujian Province, P.R. China; 5 Jianying Yan, MD. Department of Obstetrics, Fujian Maternity and Child Health Hospital, Affiliated Hospital of Fujian Medical University; No. 18, Daoshan Road, Fuzhou, 350000, Fujian Province, P.R. China

**Keywords:** Obstetric emergency hysterectomy, Subtotal hysterectomy, Total hysterectomy, Primary hospital

## Abstract

**Objectives:**

To investigate the indications of obstetric emergency hysterectomy and analyze the clinical effects of subtotal hysterectomy and total hysterectomy.

**Methods:**

We included 247 hospitalized women who had undergone abdominal hysterectomy due to obstetric reasons in Fujian Province Maternity and Child Health Hospital (a provincial class-A hospital) and Ningde People’s Hospital (a primary Class-B hospital) between January 2002 and December 2018. We identified surgical indications and clinical characteristics of the patients. Furthermore, the patients from Fujian Provincial Maternity and Child Health Hospital were subdivided into subtotal hysterectomy group and total hysterectomy group to examine general operation conditions, and postoperative complications.

**Results:**

The main surgical indications for emergency obstetric hysterectomy in Fujian Maternity and Child Health Hospital were placental implantation (49.6%) and uterine weakness (31.9%), while uterine weakness (37.5%) was the most important indication in Ningde People’s Hospital. No differences were found in operation time, hospitalization time, intraoperative blood loss, postpartum blood loss, and intraoperative fresh frozen plasma transfusion between the subtotal hysterectomy group and the total hysterectomy group. Postoperative test parameters, including postoperative prothrombin time (PT), thrombin time (TT), activated partial thromboplastin time (APTT), hemoglobin (HGB), and hematocrit (HCT), were not significantly different between the two groups. No significant difference was noted in postoperative vesicoureteral injury, pelvic hematoma, infection, and disseminated intravascular coagulation (DIC) incidence, but renal failure incidence was different (P=0.040).

**Conclusion:**

The treatment effect of subtotal hysterectomies for the cases without placenta accreta and placenta previa was similar in the two hospitals. There is no statistically significant difference in therapeutic effect between total hysterectomy and subtotal hysterectomy.

## INTRODUCTION

Obstetric emergency hysterectomy, as a method for saving patients with refractory obstetric hemorrhaging, is generally performed after 20 weeks of pregnancy and within 24 hours of fetal birth.^1^ Although treatment techniques have improved, the incidence of emergency hysterectomy (0.22%-0.62%)^2^-^4^ has not significantly decreased alongside increasing cesarean section incidence.^5^

The main indications for emergency obstetric hysterectomy are placental abnormalities, uterine weakness, and uterine rupture.^6^,^7^ Obstetric emergency hysterectomy can be subdivided into total hysterectomy and subtotal hysterectomy. Although the pros and cons of the two techniques have been investigated, whether subtotal hysterectomy should be performed prior to total hysterectomy remains controversial. This study therefore retrospectively analyzes surgical methods, surgical indications, and general conditions leading to obstetric emergency hysterectomy within different hospital categories. There is a particular paucity of data on this subject for the southeast coastal areas of China, especially for primary hospitals where refractory obstetric hemorrhage treatment is relatively limited due to resource scarcity.

We hope that this study can lay a foundation for reducing obstetric hysterectomy rates. We also hope that continuing analysis of clinical efficacy and the applicative value of subtotal hysterectomy and total hysterectomy will provide information for promoting the standardization of obstetric refractory hemorrhage diagnosis and treatment practices in primary hospitals. Finally, comparing postoperative complications and prognosis between subtotal hysterectomy and total hysterectomy patients will offer evidence for improving obstetric emergency hysterectomy practices.

## METHODS

**Fig.1:**
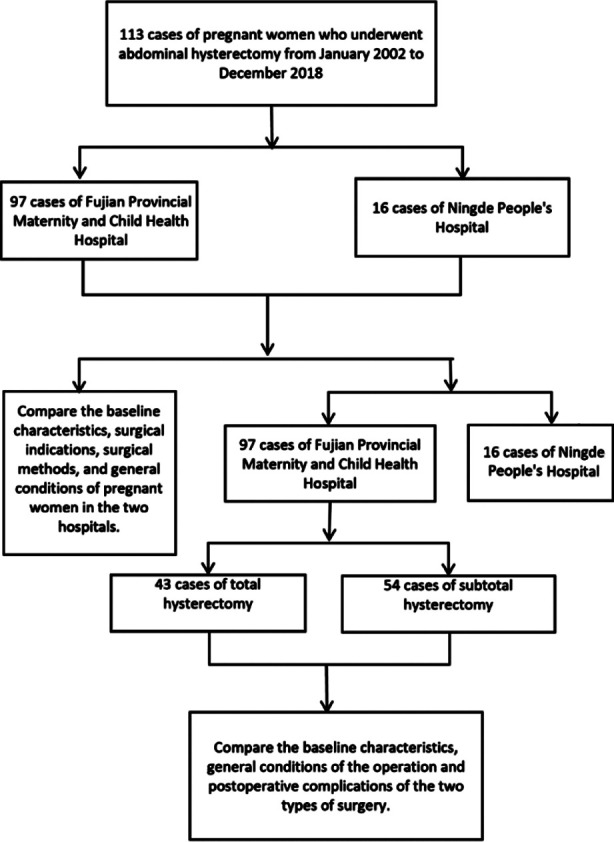
Pregnant women included in the study

This study retrospectively analyzed data on 113 pregnant women who underwent abdominal hysterectomy between January 2002 and December 2018 due to obstetric reasons. The ethics committee of our hospital approved this study on September 5^th^, 2020 (approval number 2020YJ183). Among these, 97 cases were treated at a Class-A hospital (Fujian Provincial Maternity and Child Health Hospital, while 16 cases were treated at a Class-B hospital (Ningde City People’s Hospital). Of individuals treated at a Class-A hospital, there were 54 cases of subtotal and 43 cases of total hysterectomy. All 16 patients treated at the Class-B hospital underwent subtotal hysterectomy (Fig.1).

Clinical data for included patients were collected via the electronic medical records of Fujian Maternity and Child Health Hospital and Ningde People’s Hospital. Analyzed data included: general demographic information (age, height, weight, number of pregnancies, number of births, delivery gestational week, number of abortions, number of purges, number of past cesarean sections), information on maternal complications and delivery conditions (premature membrane rupture, uterine fibroids, hypertensive disease during pregnancy, viral hepatitis, fetal position and delivery method during delivery), general operation conditions (operation time, hospitalization time, postoperative blood loss), postoperative coagulation-related indicators [prothrombin time (PT), thrombin time (TT), activated partial thromboplastin time (APTT), prothrombin activity (PTA), hemoglobin (HGB), hematocrit (HCT), platelet count (PLT)], and postoperative complications (vesicoureteral injury, pelvic hematoma, infection, disseminated intravascular coagulation (DIC), renal failure).

### Statistical Analysis

Data collection tables were constructed using Epidata 3.1 software, and SPSS 22.0 was used for data analysis. Measurement data conforming to normal distribution are represented by means ± standard deviation (*x¯*±s) and analyzed with t tests. In other situations, Mann-Whitney U tests and χ2 tests were used.

## RESULTS

Subjects were on average 31.98 ± 4.92 years of age, while average gestational age was 35.36 ± 6.08 weeks. Among the 113 cases, which included 21 transferred cases after vaginal delivery and 92 transferred cases after cesarean section, 43 cases underwent total hysterectomy and 70 cases underwent subtotal hysterectomy. Relative to subjects treated at Fujian Provincial Maternity and Child Health Hospital, pregnant women treated at Ningde City People’s Hospital are older (35.12 ± 4.69 vs 31.46 ± 4.78, P=0.005), have given birth more times (2.00 [1.00, 2.00] vs 1.00 [1.00, 1.00], P=0.001), and were more likely to have undergone uterine fibroid removal (50% vs 4.1%, P<0.001). There was no difference between the patients at the two hospitals in any other indicators (P>0.05, Table-I).

**Table I:** Patient sociodemographic data, clinical characteristics, and surgical indications.

	Ningde People’s Hospital	Fujian Provincial Maternity and Child Health Hospital	P
Age	35.12±4.69	31.46±4.78	0.005
Height (cm)	156.00±3.06	158.02±5.31	0.143
Weight (kg)	65.00±6.95	65.55±8.33	0.802
Number of pregnancies	2.50 [1.75, 3.50]	3.00 [2.00, 4.00]	0.267
Number of previous curettages	0.00 [0.00, 1.00]	1.00 [0.00, 2.00]	0.352
Number of abortions	1.00 [0.00, 1.50]	1.00 [0.00, 2.00]	0.732
Parity number	2.00 [1.00, 2.00]	1.00 [1.00, 1.00]	0.001
Gestational week (week)	39.50 [38.08, 40.00]	36.50 [33.00, 38.00]	0.002
History of enucleation of uterine fibroids (cases (%))	8 (50.0)	4 (4.1)	<0.001
Premature rupture of membranes (cases (%))	2 (12.5)	17 (17.5)	0.891
Uterine fibroids (cases (%))	8 (50.0)	18 (18.6)	0.014
Hypertension in pregnancy (cases (%))	4 (25.0)	8 (8.2)	0.115
Viral hepatitis (cases (%))	0 (0.0)	19 (19.6)	0.114
Gestational diabetes (cases (%))	0 (0.0)	15 (15.5)	0.197
Number of previous cesarean sections (cases (%))			
0 (number of cases (%))	8 (50.0)	50 (51.5)	0.829
1 (number of cases (%))	8 (50.0)	45 (46.4)	
2 (number of cases (%))	0 (0.0)	2 (2.1)	
Fetal position (cases (%))			
Cephalic presentations (number of cases (%))	10 (62.5)	68 (70.1)	0.119
Breech presentation (cases (%))	6 (37.5)	18 (18.6)	
Transverse lie (number of cases (%))	0 (0.0)	11 (11.3)	
Delivery method (number of cases (%))			
Vaginal delivery (number of cases (%))	2 (12.5)	19 (19.6)	0.743
Cesarean section (number of cases (%))	14 (87.5)	78 (80.4)	
Operation time (min)	159.75±77.13	186.13±90.46	0.294
Hospitalization time (d)	15.06±9.43	12.37±9.61	0.326
Intraoperative blood loss (ml)	2873.75±2260.80	3282.78±2815.19	0.597
Postoperative bleeding (ml)	2564.29±1895.77	2163.23±2088.49	0.650
Intraoperative red blood cell transfusion (U)	12.19±7.77	12.56±8.48	0.869
Intraoperative fresh frozen plasma (ml)	1043.75±517.65	1136.60±1106.69	0.743
PT(s)	14.23±1.92	2.19±3.29	0.001
TT(s)	13.58±2.79	25.07±37.97	0.303
APTT(s)	34.91±8.85	45.22±24.51	0.161
HGB (g/L)	86.33±18.44	79.13±14.33	0.155
HCT (%)	24.76±3.80	24.96±5.00	0.885
PLT (×10^9^/L)	124.96±80.48	152.63±92.68	0.292
Complications			0.201
Injury of vesicoureter (n)	1	4	
Pelvic hematoma (n)	1	3	
Infection (n)	1	6	
DIC(n)	2	33	

**Fig.2:**
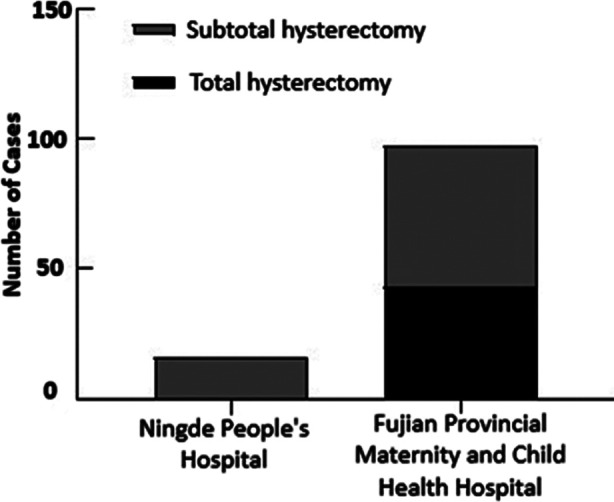
Inter-hospital comparison of surgical method selection

All patients undergoing emergency obstetric hysterectomy at Ningde People’s Hospital underwent subtotal hysterectomy. In contrast, 43 patients treated at Fujian Provincial Maternity and Child Health Hospital underwent total hysterectomy and 54 underwent subtotal hysterectomy. Fig.2 The main surgical indications for emergency obstetric hysterectomies performed at Fujian Maternity and Child Health Hospital were placenta accreta with placenta previa (38.1%), uterine contraction fatigue (31.9%), and placenta accreta without placenta previa (11.3%). Meanwhile, the main indication for procedures performed at Ningde People’s Hospital was uterine weakness (37.5%) Table-II Based on treatment location, the incidence of placenta accreta and placenta previa, as well as uterine fibroids was statistically different in the two patient populations (P<0.05).

**Table II:** Analysis of surgical indications between the two hospitals.

	Ningde People’s Hospital (16 cases)	Fujian Provincial Maternity and Child Health Hospital (97 cases)	Total	P
Placenta accreta (cases (%))	2 (12.5)	54 (55.7)	56 (49.6)	0.001
Placenta accreta and placenta previa (cases (%))	0	43 (44.3)	43 (38.1)	0.001
Placenta accreta without placenta previa (cases (%))	2 (12.5)	11 (11.3)	13 (11.5)	0.893
Uterine atony (cases (%))	6 (37.5)	30 (30.9)	36 (31.9)	0.601
Inverted uterus (cases (%))	2 (12.5)	0 (2.1)	2 (3.5)	0.036
Uterine rupture (cases (%))	2 (12.5)	6 (6.2)	8 (7.1)	0.362
Uterine fibroids (cases (%))	4 (25.0)	2 (2.1)	6 (5.3)	<0.001
Amniotic fluid embolism (cases (%))	0	5 (5.2)	5 (4.4)	0.353

There was no significant difference in operation time, hospitalization time, intraoperative blood loss, postoperative blood loss, intraoperative red blood cell infusion, or intraoperative infusion of fresh frozen plasma between procedures performed at the two hospitals (P>0.05). Moreover, postoperative hematological indexes of pregnant women at the two hospitals, including TT, APTT, HGB, HCT, PLT, were similar. Follow-up investigation of pregnant women undergoing emergency obstetric hysterectomy indicated that the difference in postoperative complications between pregnant women treated at the two hospitals was not dramatic. Table-I

Age, height, weight, parity, gestational age, number of abortions, and number of uterine evacuations were statistically different between patients undergoing subtotal and total hysterectomies at Fujian Provincial Maternity and Child Health Hospital. However, the number of pregnancies per woman in the total hysterectomy group was slightly greater than that in the subtotal hysterectomy group (4.00 [3.00, 4.50] VS. 3.00 [2.00, 4.00], P=0.020). Other indices, such as premature rupture of membranes, uterine fibroids, pregnancy-induced hypertension, viral hepatitis, the number of previous cesarean sections, fetal position at delivery, and delivery method were not statistically different between the two groups (P>0.05). However, the number of previous cesarean sections and the incidence of concurrent gestational diabetes were higher (P=0.019 and P=0.001, respectively) in the subtotal hysterectomy group relative to the total hysterectomy group. Table-III

**Table III:** The sociodemographic data, operation conditions, and postoperative complications between subtotal hysterectomy group and total hysterectomy group.

	Subtotal hysterectomy group	Total hysterectomy group	P
Age	31.37 (4.93)	31.58 (4.64)	0.830
Height (cm)	157.43 (5.50)	158.76 (5.03)	0.223
Weight (kg)	64.61 (7.31)	66.73 (9.42)	0.215
Number of pregnancies	3.00 [2.00, 4.00]	4.00 [3.00, 4.50]	0.020
Number of previous curettages	0.00 [0.00, 1.75]	1.00 [0.00, 2.00]	0.363
Number of abortions	1.00 [0.00, 2.00]	1.00 [0.00, 2.00]	0.174
Number of parities	1.00 [0.00, 1.00]	1.00 [1.00, 1.00]	0.045
Gestational week (week)	36.00 [34.00, 38.00]	37.00 [33.00, 38.00]	0.739
History of enucleation of uterine fibroids (cases (%))	1(1.9)	3(7.0)	0.207
Premature rupture of membranes (cases (%))	13(24.1)	4(9.3)	0.057
Uterine fibroids (cases (%))	11(20.4)	7(16.3)	0.607
Hypertension in pregnancy (cases (%))	6(11.1)	2(4.7)	0.251
Viral hepatitis (cases (%))	9(16.7)	10(23.3)	0.417
Gestational diabetes (cases (%))	3(5.6)	12(27.9)	0.002
Number of previous cesarean sections (cases (%))			
0 (number of cases (%))	34(63.0)	16(37.2)	0.019
1 (number of cases (%))	20(37.0)	25(58.1)	
2 (number of cases (%))	0(0.0)	2(4.7)	
Fetal position (cases (%))			
Cephalic presentations (number of cases (%))	39(72.2)	29(67.4)	0.527
Breech presentation (cases (%))	8(14.8)	10(23.3)	
Transverse lie (number of cases (%))	7(13.0)	4(9.3)	
Mode of delivery (number of cases (%))			
Vaginal delivery (number of cases (%))	8(14.8)	11(25.6)	0.184
Cesarean section (number of cases (%))	46(85.2)	32(74.4)	
Operation time (min)	170.37±74.95	180.56±89.25	0.543
Hospitalization time (days)	11.41±7.37	12.23±9.42	0.629
Intraoperative blood loss (ml)	2982.41±2416.31	3056.16±3063.95	0.895
Postpartum bleeding (ml)	2342.96±1916.17	2712.96±2001.50	0.495
Intraoperative red blood cell infusion (unit)	10.39±6.60	13.64±8.92	0.042
Intraoperative infusion of fresh frozen plasma (ml)	1118.52±1253.72	1122.09±768.45	0.987
PT(s)	21.86±32.85	19.61±29.58	0.742
TT(s)	25.07±37.97	17.05±4.16	0.182
APTT(s)	45.22±24.51	43.48±15.88	0.704
HGB (g/L)	86.33±18.44	88.67±15.83	0.515
HCT (%)	24.96±5.00	25.13±4.96	0.869
PLT (×10^9^/L)	124.96±80.48	97.69±58.47	0.068
Injury of vesicoureter (cases (%))	3 (7.0)	1 (1.9)	0.207
Pelvic hematoma (cases (%))	1 (2.3)	3 (5.6)	0.427
Infection (cases (%))	3 (7.0)	4 (7.4)	0.975
DIC (cases (%))	16 (37.2)	21 (47.7)	0.321
Renal failure (cases (%))	0	5 (19.3)	0.040
Second surgery due to bleeding (number of cases (%))	6 (14.0)	2 (3.7)	0.068

There was no visible difference between the subtotal hysterectomy group and the total hysterectomy group in terms of operation time, hospital stay, intraoperative blood loss, postpartum blood loss, and intraoperative fresh frozen plasma infusion (P>0.05, Table-III). However, the amount of red blood cell transfusions during operation was slightly higher in the total hysterectomy group (13.64±8.92 vs. 10.39±6.60, P=0.042). Moreover, no significant differences were noted in hematology indices, including PTA, PT, TT, APTT, HB, HCT, and PLT (P>0.05. Looking at postoperative complications, there was a slightly higher incidence of renal failure in the total hysterectomy group (P=0.040), but other indicators, including vesicoureteral injury, pelvic hematoma, infection, and DIC, did not differ (P>0.05, Table-III).

## DISCUSSION

This study investigated the surgical methods, surgical indications, and general conditions surrounding emergency obstetric hysterectomy operations performed in different levels of hospitals, and used this information to analyze the clinical efficacy and application value of subtotal hysterectomy and total hysterectomy. Consistent with other findings,^8^ our study found that the main surgical indications for emergency obstetric hysterectomy were placental implantation (49.6%) and uterine weakness (31.9%). Other studies have also indicated that uterine rupture is a common surgical indication for emergency perinatal hysterectomy.^9^,^10^ Indeed, our investigation of 16 cases at Ningde People’s Hospital found uterine weakness to be a primary surgical indication. Interestingly, however, this group showed no incidence of placenta accreta and placenta previa. This may be because women with a higher risk of bleeding caused by placenta accreta and placenta previa would be transferred to higher-level hospitals. Indeed, all 16 cases here were subtotal hysterectomies. Other studies have indicated that subtotal hysterectomy is more conducive to primary hospitals because the procedure is shorter and technically safer, especially for patients with elevated bleeding and adhesion of the lower part of the uterus to the bladder.^11^,^12^ Importantly, we found no differences in operating conditions and postoperative complications, indicating that primary hospital treatment quality is consistent with treatment effect achieved by the provincial tertiary hospital.

The debate between the two surgical methods for obstetric emergency hysterectomy remains controversial.^13^-^15^ A previous review indicated that subtotal hysterectomy was faster and involved less blood loss than total hysterectomy, something that was echoed in our previous study.^16^,^17^ However, here, having gathered more data over the last two years, we found in this study that there was no statistically significant difference between subtotal hysterectomy group and total hysterectomy in terms of operation time, hospital stay, intraoperative blood loss, postpartum blood loss, and intraoperative fresh frozen plasma transfusion (P> 0.05). Only red blood cell transfusion amounts were slightly elevated in the total hysterectomy group (13.64±8.92 vs. 10.39±6.60, P=0.042). Similarly, coagulation indicator analysis showed no significant difference in postoperative PT, TT, APTT, HB, or HCT between the two groups (P>0.05), with only PLT slightly elevated in the subtotal hysterectomy group (124.96±80.48 VS. 97.69±58.47). In terms of postoperative complications, we noted no significant difference in vesicoureteral injury, pelvic hematoma, infection, and DIC (P>0.05), but did see a difference in renal failure incidence (P=0.040). A similar phenomenon has been observed by other studies. A study comparing 33 cases of total hysterectomy with 69 cases of subtotal hysterectomy excluded hysterectomy as an independent risk factor for maternal death after adjusting for other factors.^3^ Similarly, other studies have found that the two surgical methods did not affect maternal mortality rate through univariate analysis,^18^ did not affect complication incidence,^4^ and did not impact urinary tract injury incidence.^19^ A cohort study further found no differences in terms of risk factors, surgical indications, surgical variables, and postoperative complications between the two methods.

As the main intervention for rescuing critically ill patients in obstetrics, performing emergency hysterectomy appropriately and accurately with strong intraoperative and postoperative management is imperative. In order to standardize diagnosis and treatment of obstetric refractory hemorrhage in primary hospitals, it is necessary to monitor pregnancies and make timely referrals for pregnant women with high-risk factors, especially concerning placental pathology. In terms of surgical selection, there is no notable difference in intraoperative and postoperative parameters for these two surgical methods. Therefore, the surgeon can choose surgical method based on personal expertise and experience, in combination with the cause of bleeding and the intraoperative situation. However, this study was only able to investigate a limited sample size, and is also hampered by being retrospective in nature. The next step is to further analyze any differences in treatment effects from different hospital categories and surgical options by expanding the sample size, collecting more diagnosis and treatment data for placenta accreta and placenta previa in primary hospitals, and extending the follow-up time for pregnant women undergoing emergency obstetric hysterectomy.

### Authors’ contributions:

**ZF & HZ:** Conceived and designed the study.

**SZ & LW:** Collected the data and performed the analysis.

**ZF & HZ:** Were involved in the Writing of the manuscript and are responsible for integrity of the study.

**JY:** Edited and made significant contributions to the study.

All authors have read and approved the final manuscript.
